# Efficacy of the refined pediatric endoscope equipment management based on the quality control mode

**DOI:** 10.12669/pjms.41.2.11368

**Published:** 2025-02

**Authors:** Shuokang Gong, Liya Wang, Xiaobo Zhao, Lijuan Zheng, Xiaohong Xi, Jiejing Dong

**Affiliations:** 1 Shuokang Gong Department of Endoscopy Room, Hebei Children’s Hospital, Shijiazhuang City, Hebei Province, 050000, China; 2 Liya Wang Department of Endoscopy Room, Hebei Children’s Hospital, Shijiazhuang City, Hebei Province, 050000, China; 3 Xiaobo Zhao Department of Endoscopy Room, Hebei Children’s Hospital, Shijiazhuang City, Hebei Province, 050000, China; 4 Lijuan Zheng Department of Endoscopy Room, Hebei Children’s Hospital, Shijiazhuang City, Hebei Province, 050000, China; 5 Xiaohong Xi Department of Endoscopy Room, Hebei Children’s Hospital, Shijiazhuang City, Hebei Province, 050000, China; 6 Jiejing Dong Department of Nursing, Hebei Children’s Hospital, Shijiazhuang City, Hebei Province, 050000, China

**Keywords:** Pediatric endoscopes, Quality control mode, Refined management

## Abstract

**Objective:**

To explore the efficacy of refined management based on the quality control (QC) mode in maintaining pediatric endoscope equipment.

**Methods:**

Eighty sets of pediatric endoscopes that were sent to the endoscopy room for cleaning, disinfection, and storage at Hebei Children’s Hospital from January 2022 to October 2023 were enrolled. Based on the maintenance method, endoscopes were divided into study group (refined management plus QC mode, n=40) and control group (conventional QC mode, n=40) using a random number table method. The rate of dirt presence, detection rate of pathogenic bacteria, adenosine triphosphate (ATP) positivity rate, cleanliness qualification rate, adverse event occurrence rate, endoscopy-related processing time, and endoscopic usage were analyzed.

**Results:**

The presence rate of dirt, detection rate of pathogenic bacteria, and ATP positivity rate in the study group were significantly lower than in the control group. The cleaning qualification rate, safety storage rate, and disinfection record qualification rate were significantly higher than those of the control group (*P*<0.05). The incidence of adverse events in the study group was lower than in the control group (*P*<0.05). The study group had significantly shorter endoscopic recovery time, packaging review time, sterilization distribution time, and clinical acceptance confirmation time than the control group (*P*<0.05). Satisfaction with endoscope use was significantly higher in the study group than in the control group (*P*<0.05).

**Conclusions:**

Refined management based on the QC mode can significantly improve the cleaning quality of pediatric endoscopes. This management mode can shorten processing time, reduce the occurrence of adverse events, and achieve high satisfaction with endoscope use.

## INTRODUCTION

With advancements in medical technology, endoscopic examinations have gradually become widely used in pediatric diseases and play an important role both in diagnostics and in guiding treatment.^1^ However, pediatric endoscopes have a complex structure, slender lumens, easy damage, and special materials, and require complex cleaning and disinfection steps. In addition, their frequent use and lack of corrosion resistance, significantly increase the difficulty of endoscopic cleaning and disinfection.^2^–^4^

The current clinical lack of unified standards makes it difficult to ensure the quality of endoscopic cleaning and disinfection, which can lead to hospital infections.^5^ Endoscope management based on the quality control (QC) mode involves extensive training of relevant personnel, refining endoscope cleaning and disinfection work, strictly standardizing the cleaning and disinfection steps, and improving the qualification rate and sterility of endoscopic disinfection.^6^,^7^ Refined management is another important clinical practice management concept based on refined, standardized, in-depth, and meticulous management. Such systematic management can ensure the standardization of various aspects of clinical work for devices and reduce the occurrence of adverse events.^8^–^10^

Current studies focus on the cleaning and disinfection quality of adult endoscopes, while the QC of children’s endoscopes is difficult, and studies on the qualified rate of sensitive indicators of refined infection control in children’s endoscopy are relatively rare. This study aimed to evaluate the clinical efficacy of a refined management model based on the QC mode using refined sensitivity indicators in maintaining pediatric endoscope equipment.

## METHODS

**Fig.1:**
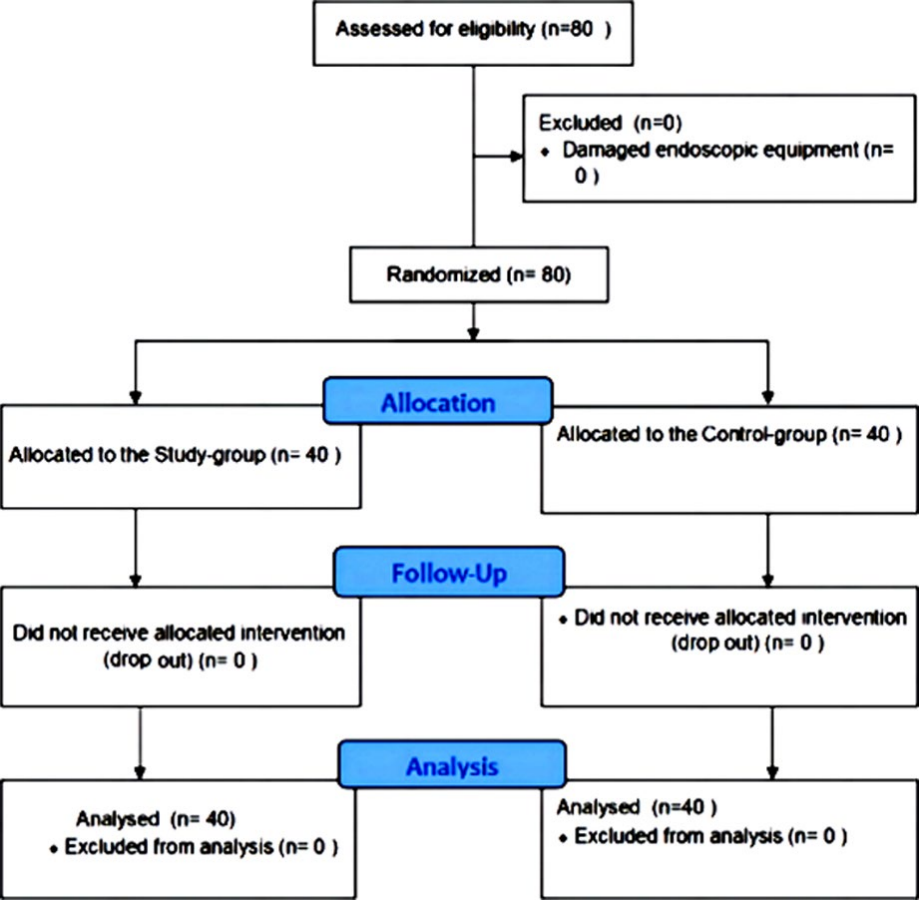
Guidelines Flow Diagram.

Eighty sets of pediatric endoscopes that were sent to the endoscopy room for cleaning, disinfection, and storage at Hebei Children’s Hospital from January 2022 to October 2023 were selected. Endoscopes were divided into two groups based on the management mode using a random number table method: the study group (refined management based on the QC mode) and the control group (conventional management based on the QC mode), with 40 sets in each group. The equipment selection flowchart was shown in Fig.1.

### Ethical Approval:

The study protocol was reviewed and approved by the Institutional Review Board of our hospital (IRB No: HRL-2023-221, Date: October 23, 2023), As this was not a human study, informed consent was waived. The study was conducted by the ethical guidelines of the 1975 Declaration of Helsinki.

### Inclusion criteria:

- All endoscopes were used and recycled in our hospital.
- Instruments that have been used by surgical patients include lumens, shafts, and grooves, which are detachable and difficult to clean.

### Exclusion criteria:

- Instrument damage prior to inclusion in the study.

### Conventional QC mode:

The routine implementation of flushing, washing, rinsing, disinfection, and drying was recorded manually. The quality of the cleaning agents and washing solutions was regularly checked. After packaging, strict inspection was performed using a magnifying glass to determine the presence of rust spots or residues on the surface of the endoscope. Flexibility of the endoscopic axis joint was checked to ensure complete and good function. Measures for cleaning unqualified instruments and monitors were reimplemented and properly recorded. The disinfection supply room management system and regulations were strictly followed to manage relevant endoscopic instruments.

### Refined management based on QC mode:

This mode including the following points: Establishment of a QC management team, staff training and management, refined endoscopic management, integration of the Hospital Information System (HIS) database server with a full endoscopic traceability system, QC process for full endoscopic traceability, and connection of the HIS system to the cleaning workstations.

A QC management team was established and included head nurses, supervisor nurses, and nurses in the disinfection supply room. Seminars were regularly organized to discuss the shortcomings of current endoscopic disinfection, cleaning, and sterilization processes and to improve management systems. A detailed division of responsibilities was established among team members for the optimization of work processes. Emphasis was placed on enhancing departmental communication, detailing work processes, and improving work quality.

Staff training and management were improved by strengthening training on relevant precautions, work steps, and workflow, enhancing basic work guidance and training, assisting personnel in improving their work skills and sense of responsibility, ensuring the smooth progress of relevant work, regularly performing personal assessments, strengthening aseptic awareness and concepts, promptly identifying existing and potential problems, and providing guidance and suggestions.

### Refined endoscopic management:

The data collector system was established and included radio Frequency Identification (RFID) card readers for endoscopic cleaning workstations, RFID card readers for cleaning mirror storage cabinet workstations, and RFID card readers for endoscopic graphic workstations. RFID card readers were installed in the leak detection, cleaning, rinsing, disinfection, final rinsing, drying, and termination stages of the endoscopic cleaning workstation. The system was able to identify the RFID card of the cleaning staff, and the endoscopic RFID card. Each decontaminator was equipped with a unique RFID card and their identity information was entered into the system. Each endoscope was equipped with a unique RFID card, and identity information such as endoscope type and body number was entered into the system. The key points for the quality of endoscopic disinfection included: the amount of cleaning solution, the amount of disinfection solution, and the operation time for leak detection, cleaning, rinsing, disinfection, final rinsing, and drying, which were entered into the system. Mandatory management was implemented: if the time did not meet the set standards, the next process could not be initiated. The cleaning workstation was equipped with speakers to indicate the start, end, or alarm of different endoscopic operations and with a computer screen to facilitate the observation and review of each operation process by the cleaning staff. The system installed RFID card readers on the endoscopic graphic workstation for reading endoscopic RFID cards during diagnosis and treatment. The system installed an RFID card reader on the endoscope cleaning storage cabinet workstation that was used to read the endoscope RFID card during the final storage.

The Hospital Information System (HIS) database server was integrated with a full endoscopic traceability system. The graphic and textual reporting workstation was opened before the endoscopic diagnosis and treatment, the endoscopic RFID card reader was identified, and the examination time, operating department, name, sex, age, phone number, hospitalization number, operating doctor, examination frequency, operating nurse, preoperative laboratory results, endoscopic diagnosis, examination equipment, home address, etc. were recorded. The endoscopic cleaning and storage workstation was connected to an endoscopic full-traceability system. The endoscope RFID card reader was identified, and the endoscope number, time, and storage environment, were recorded in real-time.

QC process for full endoscopic traceability. A system recorded each operator who swiped the card before the endoscope cleaning. Time for leak detection, cleaning, rinsing, disinfection, final rinsing, and drying of the endoscope were recorded in the system after the cleaning was completed. The RFID card for the endoscope was swiped before storing it in a clean mirror storage cabinet. The system also recorded the number and time of the endoscope used, as the endoscope RFID card was swiped every time when the endoscope was used. Cleaning, disinfection, and storage records of the endoscope were returned to the graphic and text workstation, combined with patient information and endoscopic diagnosis and treatment information for the complete multi-dimensional recording of the entire process of endoscopic diagnosis, treatment, cleaning, disinfection, storage, and use of patient information. Management of key QC points was ensured for full endoscopic traceability.

HIS system was installed on office computers and connected to cleaning workstations. The manager was responsible for inputting the decontamination IP through a browser, retrieving endoscopic decontamination traceability data, and tracking last week’s decontamination data every Monday. Any incidences of non-standard data were promptly reported to all cleaning staff for continuous improvement. Using the HIS system, the entire process of endoscopic traceability data could be retrieved, including patient personal information, operating physician information, endoscope number used, washing and disinfection process, etc. After disinfection of the endoscope, it was placed in a clean mirror storage cabinet equipped with sensors for real-time monitoring, traceability, and recording of the storage time and environment (storage temperature, wind pressure, humidity, and other parameters) of the endoscope.

### Outcome measures:

The following outcome indicators were recorded:

a) Refined sensitivity indicators for sensory control were statistically analyzed:

- The presence of dirt (the presence of blood stains, patchy stains, stains, and rust on the surface of the endoscope is evaluated as dirt);
- Pathogenic bacteria detection rate [pathogenic bacteria were isolated and identified using a fully automated bacterial (BD Phoenix™, USA) identification analyzer]. If the total number of bacteria was ≥ 20 CFU/piece, it was evaluated as unqualified);
- The positive rate of adenosine triphosphate (ATP, measured using ATP fluorescence detector (Shinva Super System 1, Zibo, China) on the treated endoscopic surface). ATP fluorescence value was detected and evaluated as positive if>10 RIU);
- Cleaning qualification rate was measured by using sterilized injection water for cleaning, testing with jelly test paper, adding color solution to the color test paper, applying it to the surface of the endoscope, joint axis, bite surface of the forceps, and lumen. If the color of the test paper does not change, it is evaluated as qualified);
- Disinfection record qualification rate (strict and accurate recording of cleaning time, and process).

b) The incidence of adverse events between the two groups, including label date errors, wet bag sampling, discrepancies between the outer label and instruments inside the bag, and incomplete instruments.
c) The time required for the two sets of endoscopy-related treatments, including the time for endoscopic recycling, packaging review, sterilization and distribution, and confirmation of clinical acceptance.
d) Physician satisfaction with the use of endoscopes, evaluated using a self-designed questionnaire from the disinfection supply center (after preliminary testing, the internal consistency reliability Cronbach’s α of this scale was 0.92, and the validity coefficient was 0.90), with a total score of 100 points (Very satisfied: ≥ 90 points, satisfied: 70-89 points, average: 60-69 points, dissatisfied: < 60 points); Total satisfaction=(Very satisfied+Satisfied) / Total number of cases × 100%.

### Statistical Analysis:

All data analyses were conducted using the SPSS software (version 25.0; IBM Corp, Armonk, NY, USA). The Shapiro–Wilk test was used to evaluate the normality of the data. The data with normal distribution were represented by mean ± standard deviation. Independent sample *t*-test was used for inter-group comparison, paired *t*-test was used for intragroup comparisons before and after comparison, and the chi-square test was used to represent the number of cases. *P*<0.05 indicated a statistically significant difference.

## RESULTS

A total of 80 sets of pediatric endoscopes were included, with 40 sets in each group. The presence rate of dirt, the detection rate of pathogenic bacteria, and the ATP positivity rate in the study group were significantly lower than those in the control group. Similarly, the cleaning qualification rate, safety storage rate, and disinfection record qualification rate were significantly higher in the study group compared to the control group (*P*<0.05) (Table-I). The incidence of adverse events in the study group was significantly lower than in the control group (*P*<0.05) (Table-II). Endoscopes in the study group had a shorter endoscopic recovery time, packaging review time, sterilization distribution time, and clinical acceptance confirmation time compared to the control group (*P*<0.05) (Table-III). Satisfaction with endoscopic use was significantly higher in the study group than in the control group (*P*<0.05) (Table-IV).

**Table-I:** Comparison of Two Groups of Sensory Control Sensitivity Indicators.

Group	Dirt presence rate	Detection rate of pathogenic bacteria	ATP positivity rate	Cleaning qualification rate	Safe storage rate	Qualified rate of disinfection records
Study group (n=40)	1(2.50)	0(0.00)	1(2.50)	39(97.50)	38(95.00)	39(97.50)
Control group (n=40)	8(20.00)	6(15.00)	9(22.50)	32(80.00)	32(80.00)	31(77.50)
χ2	4.507^a^	4.505	7.314	4.507^a^	4.114	7.314
P	0.034	0.034	0.007	0.034	0.043	0.007

a , Continuity Correction.

**Table-II:** Comparison of incidence rates of adverse events between two groups.

Group	Label date error	Spot check wet bags	External label mismatch	Lost equipment	Total incidence rate
Study group (n=40)	1(2.50)	0(0.00)	1(2.50)	0(0.00)	2(5.00)
Control group (n=40)	3(7.50)	1(2.50)	2(5.00)	2(5.00)	8(20.00)
*χ* ^2^					4.114
*P*					0.043

**Table-III:** Comparison of time spent on endoscopic related treatments between two groups (minutes).

Group	Endoscopic recycling time	Review packaging time	Sterilization distribution time	Clinical acceptance confirmation time
Study group (n=40)	2.18±0.75	3.43±1.13	2.65±0.80	3.55±1.20
Control group (n=40)	4.45±1.40	4.75±1.72	3.63±1.19	5.10±1.19
*t*	-9.092	-4.071	-4.293	-5.798
*P*	<0.001	<0.001	<0.001	<0.001

**Table-IV:** Comparison of endoscopic use between two groups.

Group	Very satisfied	Satisfied	Average	Dissatisfied	Overall satisfaction
Study group (n=40)	31(77.50)	8(20.00)	1(2.50)	0(0.00)	39(97.50)
Control group (n=40)	19(47.50)	13(32.50)	6(15.00)	2(5.00)	32(80.00)
*χ* ^2^					4.507^a^
P					0.034

a , Continuity Correction.

## DISCUSSION

The results of our study indicated that refined management based on the QC mode has high application value in the management of pediatric endoscope equipment. This management mode can improve the efficiency of endoscopic treatment and ensure the quality of cleaning and disinfection.

Xu et al.^11^ applied a refined management model to the clinical management of external medical devices in hospital disinfection supply centers and showed that this method was associated with a higher postoperative washing rate and lower incidence of non-standard packaging, wet packaging, unqualified sterilization monitoring, early release of instruments, incomplete or damaged instruments, and unqualified instrument cleaning. The incidence of missed registration, reporting, and packaging was also lower and the satisfaction score was higher in the group that was managed by the refined management model. The results of our study are consistent with the previous research. Our results suggest that establishing a management team, reasonable scheduling, and detailed division of responsibilities among team members can ensure a more scientific and standardized cleaning and disinfection process for endoscopes. It enhances the sense of responsibility of staff and can improve the quality of endoscope cleaning and disinfection.^11^,^12^ Croke 13 has shown that strict control of the cleaning and disinfection that is implemented during the refined management and includes pre-treatment, cleaning methods, soaking disinfection, and blow drying can effectively improve the compliance rate of operational details, improve the results of the endoscopic gray staining examination, and reduce the number of bacterial colonies on the surface of the sampled endoscopes.

Blázquez-Garrido et al.^14^ demonstrated that the refined management of endoscopes, encompassing classification, cleaning, disinfection, lubrication, drying treatment, inspection, and packaging of endoscopes, selection of tools, cleaning methods, special instrument cleaning measures, cleaning solution configuration, and standardized operating procedures can effectively improve the quality of endoscopic cleaning. The refined management based on the QC mode for endoscopes is instrumental in regularly checking the cleaning process and quality of endoscopes, promptly identifying misconceptions and blind spots in relevant work processes, helping to continuously improve the qualification rate of endoscopic cleaning, reducing the rate of endoscopic backwashing, effectively maintaining endoscopic instruments, extending the service life of endoscopes, and reducing the cost of instrument investment.^13^,^14^ Shin JE et al.^15^ pointed out that hospitals have a limited number of endoscopes, and their prices are relatively high. Therefore, by establishing an endoscopic QC team, strengthening training for relevant personnel, standardizing relevant cleaning and disinfection processes, refining corresponding standards, and helping to minimize the loss or damage of equipment caused by complex handover processes, refined management based on the QC mode may improve medical efficiency.^15^

The results of our study also indicate that refined management based on the QC mode can reduce the occurrence of adverse events and improve device satisfaction. It is plausible that this management model can improve the quality of endoscopic cleaning and disinfection, ensuring clinical efficacy and accuracy and resulting in higher satisfaction. Chhabria et al.^16^ have shown that implementing refined management of endoscopes improves the satisfaction of the disinfection supply center staff, the standardization of operating procedures, optimizes endoscope recycling, disassembly, classification, and disinfection, improves work effectiveness and safety, and ensures medical and patient safety. Related studies have also confirmed that efficacy of refined management which can reduce the incidence of illegal operation of medical equipment in endoscopy centers, failure to fill-in records as required, lower the incidence of accidents, ensuring clinical satisfaction.^17^–^20^ This is consistent with the conclusions of our study.

### Strengths and limitations:

The current study has certain strengths. First, it fills the research gap that there are relatively rare studies on the qualified rate of sensitive indicators of refined infection control in children’s endoscopy. Second, to explore the efficacy of refined management based on the QC mode in maintaining pediatric endoscope equipment, which is helpful to standardize the homogenized cleaning and disinfection operation process of children’s endoscopes, improve the cleaning and disinfection quality, record authenticity and accuracy, improve the turnover rate of endoscopes, and reduce the proportion of decontaminating personnel. The study also has several limitations. First, it is a single-center study with a small sample size. Second, only pediatric endoscopes were included. Further studies are needed to verify the effect of the management mode on the disinfection of the equipment used for duodenoscopy. Third, due to the full involvement of the management team in equipment management, blind methods could not be used in this study. Large-sample, multicenter studies are needed to overcome the existing design flaws.

## CONCLUSION

Refined management based on the QC mode can significantly improve the cleaning quality of pediatric endoscopes. This management mode can shorten processing time, reduce the occurrence of adverse events, and achieve high physician satisfaction in clinical practice.

### Author’s Contributions:

**SG:** Study design, literature search, manuscript writing, manuscript revision, validation and is responsible for the integrity of the study.

**LW, XZ, LZ, XX and JD:** Data collection, data analysis, interpretation and critical review.

All authors have read and approved the final manuscript.
